# Interaction between forest biodiversity and people’s use of forest resources in Roviana, Solomon Islands: implications for biocultural conservation under socioeconomic changes

**DOI:** 10.1186/1746-4269-10-10

**Published:** 2014-01-27

**Authors:** Takuro Furusawa, Myknee Qusa Sirikolo, Masatoshi Sasaoka, Ryutaro Ohtsuka

**Affiliations:** 1 Graduate School of Asian and African Area Studies, Kyoto University, Room #AA431, Research Bldg. No. 2, Yoshida-Honmachi, Sakyo-ku, Kyoto 606-8501, Japan; 2 National Herbarium & Botanical Gardens, Ministry of Forestry, Honiara, Solomon Islands; 3 Graduate School of Letters, Hokkaido University, Kita 10, Nishi 7, Kita-ku, Sapporo 060-0810, Japan; 4 Japan Wildlife Research Center (JWRC), 3-3-7 Kotobashi, Sumida-ku, Tokyo, Japan

**Keywords:** Biocultural diversity, New Georgia Island, Quantitative ethnobotany, Roviana, Socioecological production landscape (SEPL), Traditional environmental knowledge (TEK), Solomon Islands

## Abstract

**Background:**

In Solomon Islands, forests have provided people with ecological services while being affected by human use and protection. This study used a quantitative ethnobotanical analysis to explore the society–forest interaction and its transformation in Roviana, Solomon Islands. We compared local plant and land uses between a rural village and urbanized village. Special attention was paid to how local people depend on biodiversity and how traditional human modifications of forest contribute to biodiversity conservation.

**Methods:**

After defining locally recognized land-use classes, vegetation surveys were conducted in seven forest classes. For detailed observations of daily plant uses, 15 and 17 households were randomly selected in the rural and urban villages, respectively. We quantitatively documented the plant species that were used as food, medicine, building materials, and tools.

**Results:**

The vegetation survey revealed that each local forest class represented a different vegetative community with relatively low similarity between communities. Although commercial logging operations and agriculture were both prohibited in the customary nature reserve, local people were allowed to cut down trees for their personal use and to take several types of non-timber forest products. Useful trees were found at high frequencies in the barrier island’s primary forest (68.4%) and the main island’s reserve (68.3%). Various useful tree species were found only in the reserve forest and seldom available in the urban village. In the rural village, customary governance and control over the use of forest resources by the local people still functioned.

**Conclusions:**

Human modifications of the forest created unique vegetation communities, thus increasing biodiversity overall. Each type of forest had different species that varied in their levels of importance to the local subsistence lifestyle, and the villagers’ behaviors, such as respect for forest reserves and the semidomestication of some species, contributed to conserving diversity. Urbanization threatened this human–forest interaction. Although the status of biodiversity in human-modified landscapes is not fully understood, this study suggested that traditional human modifications can positively affect biodiversity and that conservation programs should incorporate traditional uses of landscapes to be successful.

## Background

Solomon Islands is a high-priority area for biodiversity conservation because of its location in the east Melanesian Islands biodiversity hotspot [1,2]. The rich biological diversity there stems from the fact that its more than 900 islands, covering 28,400 km^2^, have never been in land contact with the Asian continent or New Guinea Island, allowing a unique tropical rainforest flora and fauna to evolve [2]. The biodiversity is also perceived to be due to a lack of human intervention, but archaeological and forest ecological studies have indicated that the ecosystem is actually composed of many very old forests that had once been cleared by ancestral people in the Western Solomon Islands [3]. These societies, as with other societies in tropical rainforests, depended on forest ecosystem services for their traditional subsistence, including agriculture, the collection of natural resource products, fishing, and hunting [4,5], and also had a spiritual connection to nature [6], and consequently learned to use the forest resources sustainably [7-9]. These traditional societies have often contributed to, rather than hindered, the creation and conservation of biodiversity, but such ‘positive’ human impacts are methodologically difficult to study and have often been neglected or undervalued in conservation programs.

Although various efforts have been made to record and analyze the biocultural diversity in Solomon Islands and other Melanesian and Southeast Asian societies [7-11], quantitative data are lacking. Recent studies and global initiatives (e.g., Convention on Biological Diversity) have highlighted the importance of integrating biodiversity conservation with the rights of indigenous people to use their ecosystems to improve their lifestyles [12-15]. In contrast, some top-down conservation decisions by agents outside of the communities have ignored local needs, causing disputes with local people and achieving limited success. Proponents have insisted that biodiversity conservation should aim to preserve sustainable human-modified natural environments, also called social–ecological production landscapes (SEPL), by encouraging broader global recognition of their value (e.g., Target 3 in Strategic Plan for Biodiversity 2011–20 and SATOYAMA Initiative in the 10th Meeting of the Convention of the Parties, Convention on Biological Diversity (CBD-COP10), 2010) [16-18]. Descriptive reports of such SEPLs [6,14,17] have noted that forest consists of patches (including fallow forest and sacred forest) and that zoning of protected forest and agricultural land may decrease patch diversity [14].

Recently, several academic and non-governmental projects have worked to establish community-based nature conservation. In Solomon Islands, 87% of the land is classified as ‘Customary Land’ and is managed by traditional genealogical groups [19], so that rural communities themselves have autonomy over their forests. Consensus among the Customary Land members and integration of traditional ecological knowledge are recognized as key factors for successful conservation [20-22]. However, human impacts have recently increased in Solomon Islands, causing deterioration of biodiversity via population increases and socioeconomic globalization [5,23-25], e.g., by the expansion of agricultural lands, commercial logging, or the building of timber and oil palm plantations and urban infrastructure. The people’s demand for commodities has increased with their recent exposure to Western culture and continues to increase rapidly. In addition, the export of natural resources has been a main source of cash income in the country, so control of resource development has been limited [19,24,26]. In urbanized areas, land disputes have hindered consensus for Customary Lands protection, allowing forest exploitation to continue [27]. To avoid overexploitation of forest resources, ecological services must satisfy the society’s needs, and the society must recognize this to achieve consensus. However, how much ecosystem services were traditionally received by the people and what is lost to overexploitation is not clear.

In this study, we employed an ethnobotanical approach [28,29] combined with vegetation surveys in two villages, one rural and one urbanized, to analyze the human–biodiversity relationships in the Western Solomon Islands. Our main objectives were to address: (1) how the people use different plant resources from forest and landscape diversity, (2) whether and how traditional ways of subsistence may have contributed to the creation and conservation of forest biodiversity, (3) how recent socioeconomic changes (i.e., urbanization) could impact this diversity, even in communities with autonomy over the forests, and (4) how biodiversity conservation programs can integrate local peoples’ modifications of forests. This region of Solomon Islands is ideal for such a study because it has existed as a largely traditional society until recently and is still in the early stages of modernization. Special attention was paid to documenting the types of landscapes and how the behavior of the local people affected biodiversity. In addition, we studied how such interrelationships are affected by recent socioeconomic changes.

## Methods

### Study sites

**Figure 1:**
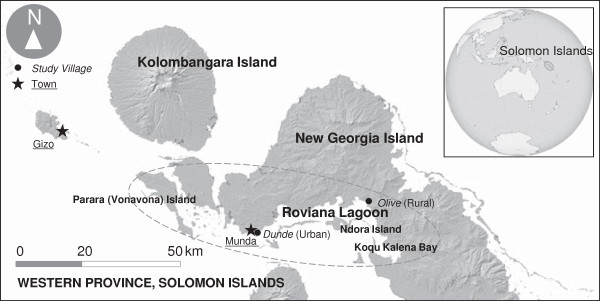
**Locations of the study villages in the Roviana region, Solomon Islands.** Map obtained from the USGS (2004). The global inset map was obtained from Wikipedia commons.

This study was conducted in the Olive and Dunde villages in the Roviana region, Western Province, Solomon Islands (Figure 1). Roviana is located in the southwestern region of New Georgia Island and includes the nearby barrier islands, with an area extending 150 km from Koqu Kalena Bay to Munda. Almost all inhabitants in this area (pop. 14,805) speak the Roviana language and share the same ancestors and similar cultures, social institutions, and ecological conditions [30,31]. Munda, the fourth largest town in the country, is a commercial center with governmental stations and several villages.

Dunde village, the largest settlement in the Munda area, was chosen as the urban study site. Olive village was chosen as the rural study site; it is one of the largest villages in the Saikile Customary Land and is approximately 32 km east of Munda, without roads or public transportation. The ‘urban’ Solomon Islanders are still very tied to their land base and often exhibit a degree of subsistence consumption, so that they are actually only semi-urban. Olive is much less affected by modern life. For instance, in Dunde with Olive, 86% and 28% of the houses are built in a modern style, 93% and 17% of households have rainwater tanks, 33% and 11% of households have outboard motors, and the average monthly household incomes are 1,752 SBD (Solomon Islands Dollars) and 378 SBD, respectively. However, there is a large amount of socioeconomic variation, especially in urban areas, as we reported elsewhere [32,33]; electricity and water lines are available only in Dunde. Regardless of these large economic differences, oral history and archaeological evidence suggest that the Dunde and Olive people are descended from a single ancestral population that migrated from Nusa Roviana Island, a small island located near Munda, in the late 18th or 19th century [34-36]. In addition, ecological conditions are similar throughout the Roviana region.

The most important form of subsistence agriculture in the Roviana region is the shifting cultivation of tuberous crops, in which lands are rotated between cultivation and fallow ground. We previously reported that this shifting cultivation is sustainable, with sufficient productivity to allow for an appropriate fallow period in Olive [25,33]. However, with Dunde’s increased population and commercialization, the crop rotation cycles have been shortened, and the land has become unproductive [33]. Forest has also been used as a source of various kinds of resources, such as building materials, medicinal plants, and tools, and for magic/ritual purposes.

Since the arrival of the first Christian mission at the end of the 19th century, the Roviana people have gradually converted, and now almost all are Christian. However, traditional ways of thinking and behaving are still practiced in daily life. In addition, in Dunde, the coastal areas have been settled and converted to township functions; the construction of infrastructure began during the European colonial period (late 19th century) and accelerated during World War II, when both Japan and the United States built airfields and bases. Selective commercial logging began in the Western Province in the 1960s and in the Roviana Lagoon area in the 1980s [19,37]. In the Saikile Customary Land, areas near Olive village were logged in 1993–94. While the logging negatively affected the forest, the local people profited from employment, royalty payments, and improved infrastructure. The forests surrounding the Dunde area were not logged because the rights to those forests were violently disputed within and among communities and clans [27]. However, the people have been economically affected by logging operations in nearby areas (e.g., Vonavona, Enoghae) since the 1960s through employment and royalty payments. One of the authors (TF) has lived in Roviana for a total of approximately 2 years since 2001, speaks the local Roviana language, understands the local customs and culture, and has built a rapport with the people [25,32,33,37,38].

### Local landscape interviews

The authors (TF, MS, and RO) walked throughout the territorial forests, gardens, plantations, and other subsistence lands. During this participatory observation, we classified the landscape. Forest ecological and floristic surveys [39] and plant resource surveys [4] of the whole Solomon Islands are helpful to understand the landscape and vegetation, and ethnoecological studies in the neighboring Marovo region are also informative [5,40]. In addition, brief descriptions of the Roviana landscape are available [41]. However, this information was not sufficient to describe the human–forest relationships, so we evaluated the landscape and vegetation of the study sites using the following methods.

**Figure 2:**
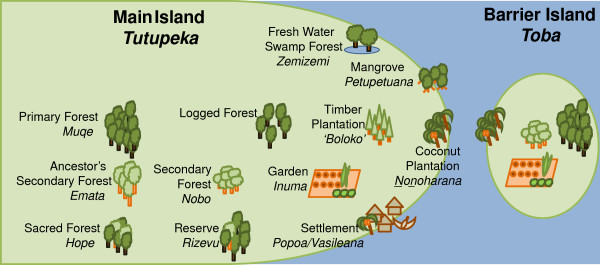
Forest and land use classifications in the local Roviana language.

Locally recognized land-use classes were identified through interviews with four local elders who had been recommended as forest experts by a committee of leaders. The authors visited various locations with these experts, who identified local Roviana names for different land use classes, including a variety of forested classes (hereafter called ‘forest classes’). In our protocol, any disagreements were to be resolved by discussion among the experts, authors, and other villagers, although such disagreements rarely happened. The ecological characteristics of 12 land-use classes on New Georgia Island were also determined during these interviews (Figure 2). Four of the 12 land use classes were found on the barrier islands (e.g., Ndora Island, an extension of Olive). The villagers used terms *tutupeka* and *toba* for the geographic characteristics of New Georgia Island and the barrier islands, respectively. TF observed and participated in various subsistence activities and confirmed that these classes were widely recognized and frequently referenced in daily life; although the number of experts (4) was small, the classifications reflected widespread recognition by the villagers.

### Vegetation surveys

Quadrat vegetation surveys were conducted in seven forest classes: (1) primary forest (*muqe*) on the main island, (2) primary forest on the barrier island, (3) secondary forest (*nobo*, 15-year-old fallow land) on the main island, (4) secondary forest (15-year-old fallow land) on the barrier island, (5) reserve forest (*rizevu*) on the main island, (6) selectively and commercially logged forest (originally primary forest; 8–9 years after operation) on the main island, and (7) mangrove forest (*petupetuana*). Freshwater swamp forest (*zemizemi*) and coastal vegetation were also surveyed but not analyzed in this study. The ancestor’s secondary forest (*emata*) and sacred forest (*hope*) were excluded from the survey because these classes, existing as patches in primary or secondary forests, were small or difficult to distinguish independently of the experts. Settlement vegetation (*popoa/vasileana*) was also excluded because of the logistic challenges to establishing quadrats in privately-managed plots.

Four 25 × 25-m quadrats were established in each forest class. i.e., 0.25 ha in total per class. Small plots can been problematic in vegetation surveys, but 0.25 ha quadrats or smaller have been used successfully when locally-defined forest types were not extensive enough to establish larger plots [42]. Because the local people near the rural village recognized two separate areas as reserve forests, four quadrats were established in each of these two forests: i.e., 0.5 ha in total for this class only. For the statistical analysis, the number of trees counted in the reserve forests was divided in half so that all forest types were comparable. The vegetation surveys were conducted in the territory of the Saikile Customary Land. All trees > 10 cm in DBH (diameter at breast height) were counted and identified with the local Roviana name by the experts. If the experts did not know or could not agree on a name, the plant was listed as ‘unknown’. Botanical specimens were collected, stored in liquid alcohol in the field, and later dried at Munda Forestry Station, Ministry of Forestry of the Solomon Islands Government.

### Plant use observations

For detailed observations of plant use, 15 and 17 households were randomly selected from the 64 and 206 households in Olive and Dunde, respectively. Written informed consent for participation in the survey was obtained from each head-of-household (head). The participants were informed that they could withdraw from the study at any time and that they had a right to refuse to answer any questions. All interviews were conducted in the local Roviana language by TF with the help of local assistants (Mr. Edwin Huti and Mr. Rex Daga). No economic incentives were provided to the participant households to avoid bias; however, following the completion of the research, a suitable cultural gift (food) was given to each household.

#### *Food*

All foods consumed in each study household during a 1-week period were observed and their weights were recorded every 80–90 minutes from 7:00 a.m. to 8:20–8:30 p.m. every day. When a participant ate food outside the village, the diet information was obtained through an interview. The energy (MJ) contribution of the plants was estimated using measurements and interviews in tandem with food composition tables [43,44]. The food surveys were conducted in August in both villages to avoid seasonal differences between the two villages.

#### *Building materials*

TF visited every household and asked the head and/or builder to provide the local Roviana name for each material used during construction, i.e., the names of the materials used for floors, walls, poles, rafters, beams, roofs, and other parts of the houses.

#### *Medicine*

Each household was visited daily, and the head and/or spouse were asked to report any illnesses and describe the treatments, including medicinal plants, used in that household every evening for 28 days. When the patient consulted other villagers or herbalists outside of the village, the herbalist was interviewed about the recipe. If the herbalist refused to disclose his/her recipe, that treatment was excluded from the analysis. We tallied the total number of ill person-days and the numbers of ill person-days on which traditional and Western medicines were used. On some days, the ill person used both traditional and Western treatments.

#### *Tools*

Tools made from plants were observed and surveyed in interviews. First, the head and/or spouse were asked to list all plant-made tools owned. A single, village-wide list of tools was compiled by visiting each household in turn. Then, each household was visited again, and the heads/spouses were asked what materials were used for each item on the final list (27 types of tools). Note that any plants used for rituals and magic, which are now rarely observed, were categorized as tools.

### Botanical name identification

As described above, the forest experts identified the local Roviana names of plants found during the vegetation surveys, and the household heads, adult members, and/or house builders identified the names of plants used for food, building materials, medicines, or tools. Living plant specimens were shown to the interviewees during the household surveys, if necessary. Botanical specimens were collected in the field, and one of the authors (MQS) identified the scientific names at the Poitete Institute of Forestry, Western Province, Solomon Islands. The local Roviana names were used as species designations in analyses to connect the vegetation and plant use data, although, in some cases, the villagers recognized two or more taxonomic species as one Roviana vernacular name or *vice versa*. English names were used for convenience.

### Statistical analysis

The similarity of species composition between each pair of forest classes was calculated using the Sørensen–Dice similarity index:

$$\mathrm{Sorensen}-\mathrm{Dice}\;\mathrm{similarity}\;\mathrm{index}\;\left(\%\right)=2c/\left(a+b\right)\times 100\%$$

where *c* is the number of species observed in both forest classes and *a* and *b* are the number of species in forest classes A and B, respectively. This index is useful for assessing ecological community data and is sensitive to heterogeneous data [45]. The proportions of useful trees in each vegetation quadrat were used to compare plant use in Dunde and Olive using the *χ*-square test. All statistical analyses were performed using the R version 2.15 (R Foundation for Statistical Computing, Vienna, Austria).

## Results

### Folk landscape and vegetation

Figure 2 shows the vegetation and land use classes recognized by the local people. The local people call the geographic setting of New Georgia Island *tutupeka* and that of the barrier islands *toba*. In their understanding and in the results of scientific surveys [27], the barrier island’s soil and vegetation are different from those of the main island. Land systems in New Georgia are characterized by dominant canopy species, such as *Calophyllum vitiense* Turr., *C. kajewskii* A. C. Smith, *Dillenia* spp.. and *Campnosperma brevipetiolata* Volk. in lowland forests and *Terminalia brassii* Ewell. in swamps, while the barrier islands are dominated by *Pometia pinnata* J. R. Forst. and G. Forst. and *Vitex cofassus* Reinw. ex Blume.

Primary forests—i.e., those recognized as being unmodified by humans—are called *muqe*. In reality, the *muqe* are used by the people to collect non-timber forest materials or to hunt wild pigs and are not pristine but rather experience human–forest interactions. In addition, Bayliss-Smith et al. [3] reported that ‘primary’ forests on New Georgia Island may actually include sites of former settlements, forest clearings, and agriculture (irrigated taro terraces called *ruta*).

One type of old secondary forest is called *emata*. This forest type is called an ancestor’s secondary forest because it was deforested and abandoned before the current people were born. According to the interviewees, some gigantic trees with high customary value, such as *Canarium salomonense* B. L. Burtt, are characteristic in such forests. The *emata* is sometimes recognized as providing evidence of their ancestor’s customary ownership of that land [5,35,36]. Meanwhile, forest areas previously inhabited by ancestors or used for rituals are now considered shrines—called *hope*—and entry and tree felling are prohibited there. The people believe that trespassers will be punished by supernatural powers (*tomate*). These sacred forests represent an aspect of the people’s customary land management.

Fallow or secondary forests that had been abandoned after shifting cultivation are called *nobo*. According to the interviewees, the residents gather medicinal plants and small trees and palms for building houses in the *nobo*. In our vegetation surveys, the secondary forest was characterized by a number of *Macaranga* tree species. In addition, on the main island, the existence of several *Commersonia bartramia* (L.) Merr. and a pioneer species, white beech (*Gmelina moluccana* Backer ex K. Heyne), was characteristic. On the barrier islands, *Syzygium* spp. and *Flueggea flexuosa* Müll. Arg. were common.

Logged forest is now a prevalent type of forest on New Georgia Island, although it has no Roviana name. The major target is upper canopy species with commercial value, such as *Calophyllum* spp., *P. pinnata*, *Dillenia salomonensis* (C. T. White) Hoogl., *Terminalia brassii* Exell, and *V. cofassus*. Some species, such as white beech, are protected by agreements between the local people and logging companies. In addition, felling trees of < 60 cm DBH is prohibited by forestry acts, as is logging near rivers or flowing streams and on steep hills.

Although reforestation for timber plantations commenced in the 1990s, this activity intensified in the 2000s [37,46]. The logged forest was clear cut to create lands for timber plantations. Such community-level reforestation has accelerated under the leadership of one of the largest church groups (Christian Fellowship Church: CFC) in New Georgia Island. Teak (*Tectona grandis* L. f.) and rainbow eucalyptus (*Eucalyptus deglupta* Blume) are common plantation species. In addition, oil palm (*Elaeis guineensis* Jacq.) plantations have expanded in the northern part of New Georgia but only to a limited extent in Roviana during the study period. Although large-scale logging operations are not conducted in the urban territory, expectations of future cash income have driven the people to clear-cut the secondary forest for timber plantations.

There are reserve forests called *rizevu* (borrowed from the English word ‘reserve’) near the rural village. These nature reserves were established in accordance with the recommendations of local chiefs and CFC leaders. Although logging by multinational companies and agricultural cultivation are both prohibited in these areas, the local people are allowed to cut down trees for their personal use and to remove several non-timber forest products. The vegetation in these reserve forests differs from that in both primary and secondary forests because of continuous anthropogenic impacts. These forests are characterized by a mixture of climax (*C. kajewskii*) and pioneer (*Palaquium erythrospermum* H. J. Lam., *Garcinia celebica* L., *Elaeocarpus floribundus* Blume, and *C. brevipetiolata*) species. The reserve forests are found in the rural, not urban, areas.

Mangroves (*petupetuana*) provide not only plant resources but also hunting grounds for shellfish and crabs and are important to the rural people. For example, the interviewees insisted that a group of people once protested and forced a logging company to halt operations when an inflow of red soil decreased the populations of shellfish and crabs. The dominant species is *Bruguiera gymnorhiza* (L.) Lam., followed by *Rhizophora apiculata* Blume. In contrast, mangroves are quite rare in urban villages because the coastal area has been converted to infrastructure and settlements.

The primary crops are sweet potatoes (*Ipomoea batatas* (L.) Lam.) and cassavas (*Manihot esculenta* Crantz); traditional tuberous crops, such as taro (*Colocasia esculenta* (L.) Schott) and yams (*Dioscorea* spp.), are also planted in approximately half of the gardens. Non-edible plants, such as *Coleus* spp., are also planted for decoration or magic (e.g., protecting crops from pest animals) in gardens. Settlements are called *popoa* or *vasileana*. Almost all trees and palms that grow in the settlements were either domesticated or semidomesticated and used for various purposes. For instance, all trees of > 10 cm DBH found along a road in Olive village were either coconut (*Cocos nucifera* L.), betel nut (*Areca catechu* L.), cut nut (*Barringtonia procera* (Miers) R. Knuth), tropical almond (*Terminalia catappa* L.), or kapok (*Ceiba pentandra* (L.) Gaertn. and *Bombax malabaricum* DC.). Introduced flowering plants, such as *Catharanthus roseus* (L.) G. Don., were planted in some houses for ornamental purposes.

### Plants used in daily life

#### *Plants used for food*

Based on the household survey, 149 species were used for food, medicine, building materials, or tools during the study period; (a list of all plants used is available as Table 1) 39 of these species were used as food. Sweet potato and cassava were the primary sources of energy in both villages (38.7% and 18.3% in the rural and urban villages, respectively). Six representative tree or palm species that provided the next highest levels of energy are shown in Table 2. Coconut contributed to approximately 4% of the total energy intake in both villages. Canarium nuts (*Canarium indicum* L. and *C. salomonense*) amounted for 3.3% of energy intake in the rural area but only 0.1% of energy intake in the urban area. While most of the food plants were cultivated or planted in gardens, gnetum tree (*Gnetum gnemon* L.) grew in *nobo* secondary forest; the people did not always plant this tree but usually refrained from weeding/hurting it under semidomesticated conditions (i.e., they have not been fully domesticated but are protected by the people in the wild and near settlements or gardens [36,37]). Fruits were collected from wild large-leafed mangrove (*B. gymnorhiza* (L.) Lam.).

**Table 1:** List of plants used in the Roviana, sorted alphabetically by the Roviana name, with use purposes observed

**Roviana name**	**Scientific name**	**Family**	**Plant type **^**a**^	**Use purpose **^**b**^	**Forest class **^**c**^
*Agana*	*Pandanus* spp.	Pandanaceae	pl/tr	T	MM
*Agana pinomo*	*Pandanus* sp.	Pandanaceae	pl/tr	T	
*Aroso*	*Calamu*s spp.	Arecaceae	cl/pl	B, T	
*Aroso inoko*	*Calamus* sp.	Arecaceae	cl/pl	B	
*Asama*	*Lygodium* spp.	Schizaeaceae	fn/cl	T	
*Babageva masa*	*Heritiera littoralis* Ait.	Sterculiaceae	tr	T	MR, MM
*Balusa*	*Ochroma pyramidale* Urb.	Bombacaceae	tr-l	T	
*Bebea*	*Tournefortia argentea* L.	Boraginaceae	sh	T	
*Bekoto*	Various small palms	Arecaceae	pl	B	
*Beti*	*Bambusa* spp.	Poaceae	gr/tr-s	B, T	
*Binisi*	*Phaseolus vulgaris* L.	Fabaceae	hb	F	
*Binisi noki*	*Trichosanthes cucumerina* L.	Cucurbitaceae	hb/cl	F	
*Bobogele*	*Pemphis acidula* J.R. & G. Forst.	Lythrales	sh/tr-s	B, T	
*Bobopa*	*Epipremnum* sp.	Araceae	cl	M	
*Bolava*	*Haplolobus canarioides* Leenh.	Burseraceae	tr	B	MP, MR, ML
*Bosi*	*Euodia salomonensis* Merr. & Perry	Rutaceae	tr	B	MR, MS, BS
*Bosi suka*	*Euodia elleryana* Muell.	Rutaceae	tr	B	MR, MS
*Bou*	*Fagraea gracilipes* A. Gray	Rubiaceae	tr	B, T	
*Buni*	*Calophyllum* spp.	Clusiaceae	tr	B, T	MP, MR, ML
*Dadao*	*Barringtonia asiatica* (L.) Kurz	Lecythidaceae	tr	T	
*Dalou*	*Pandanus* sp.	Pandanaceae	pl/tr	T	
*Deri*	*Citrullus lanatus* (Thunb.) Mansf.	Cucurbitaceae	hb/cr	F	
*Dikidiki*	*Dioscorea esculenta* (Lour.) Burk.	Dioscoreaceae	hb/cl	F	
*Dodoru*	*Trema orientalis* (L.) Bl.	Ulmaceae	tr	B	MS
*Domu*	Unidentified		tr	B	
*Edeve*	*Metroxylon* spp.	Arecaceae	pl	(F), B, T	
*Eehara*	*Horsfieldia spicata* (Roxb.) Sinclair	Myristicaceae	tr	B	MP, MR, ML, BP, BS
*Egipalanti*	*Solanum melongena* L.	Solanaceae	hb/sh	F	
*Elelo bakua*	*Cassia alata* L.	Caesalpiniaceae	sh	M	
*Elohilu*	Unidentified		tr	B	
*Gaekubo*	*Garcinia celebica* L.*, G. solomonensis* A.C. Sm.	Clusiaceae	tr	B	MP, MR, MS, ML
*Geholo*	*Schleinitzia novo-guineensis* (Warb.) Verdc.	Leguminoceae	tr-s	B	
*Gozigolo*	*Scindapsus altissimus* V.A.V.R.	Araceae	cl	T	
*Guava*	*Psidium guajava* L.	Myrtaceae	tr-s	F, T	
*Gurata*	*Morinda citrifolia* L.	Rubiaceae	tr-s	M	
*Habe*	Unidentified			T	
*Haila*	*Syzygium* sp.	Myrtaceae	tr	T	BP
*Hakua*	*Musa* spp.	Musaceae	hb/tr-s	F	
*Halagire*	Pandanaceae			T	BS
*Hame*	*Calophyllum paludosum* C.T. White., *C. neo-ebudicum* Guill.	Clusiaceae	tr	B	MR
*Harekete*	*Microsorium scolopendria* (Burm. f.) Copel.	Polypodiaceae	fn/cl	M	
*Hebere*	*Dillenia ingens* Burtt	Dilleniaceae	tr-l	B	MR, MS, ML
*Heta (heta manavasa)*	*Areca catechu* L.	Arecaceae	pl	F, M, B	
*Heta pinomo*	*Areca macrocalyx* Zipp. Ex Bl.	Arecaceae	pl	(F), B	
*Hioko*	*Syzygium* sp.	Myrtaceae	tr	B	MP, MR, MS, BP, BS
*Hipahipala*	*Syzygium* sp.	Myrtaceae	tr	B	
*Horehore*	*Calophyllum* sp.	Clusiaceae	tr/tr-l	B	MP, MR, MS, ML
*Hovaka*	*Burckella obovata* (Forst.) Pierre	Sapotaceae	tr-l	B, T	
*Igisi*	*Piper betle* L.	Piperaceae	sh/cl	F, M	
*Ika pengi*	Unidentified			B	
*Ivili*	*Intsia bijuga* (Colebr.) Kuntze	Caesalpiniaceae	tr-m	B, T	
*Kabisi*	*Brassica chinensis* L.	Cruciferae	hb	F	
*Kakarumu*	*Lumnitzera littorea* (Jack.) Voigt	Combretaceae	tr-s	B, T	
*Ka* ***n*** *ana*	*Gulubia macrospadix* (Burret) H.E. Moore	Arecaceae	pl	B	MP, MR, MS, ML
*Kapuhu*	*Dillenia salomonensis* (C.T. White) Hoogl.	Dilleniaceae	tr	B, T	MRML
*Karuvera*	*Alocacia macrorrhiza* (L.) G. Don	Araceae	hb		
*Keto*	*Zea mays* L.	Poaceae	gr/hb	F	
*Kikilapa (kilala)*	*Ochroma pyramidale* Urb.	Bombaceae	tr-l	T	
*Kinu*	*Barringtonia procera* (Miers) R.Knuth	Lecythidaceae	tr	F	
*Kokeqolo*	*Aglaia brassii* Merr. and Perry	Meliaceae	tr	B	MP, MR, ML, BP, BS
*Kosikosiri*	*Diplazium esculentum* (Retz.) Sw.	Athyriaceae	fn	F	
*Kukaba*	*Cucumis sativus* L.	Cucurbitaceae	hb/cl	F	
*Kureu*	*Diospyros ferrea* (Willd.) Bakh.	Ebenaceae	tr-s	B	MP, MR
*Laini*	*Citrus aurantifolia* (Christm.) Swing.	Rutaceae	tr-s	F, M	
*Lemone*	*Citrus limon* (L.) Burm. f.	Rutaceae	tr-s	(F), M	
*Leqe*	*Gnetum gnemon* L.	Gnetaceae	tr	F	BS
*Levaleva*	Unidentified		tr	B	ML
*Likisi*	*Allium porrum* L.	Liliaceae	hb	F	
*Liqeliqe*	Unidentified		tr	B	ML, BP
*Lozi*	*Ceiba pentandra* (L.) Gaertn.*, Bombax malabaricum* DC.	Bombaceae	tr-m	T	
*Lulua*	*Amoora cucullata* Roxb.	Meliaceae	tr	B	MP, MR, BP, BS
*Luluzu*	*Mikania cordata* (Burm. f.) B.L. Rob.	Asteraceae	hb/cl	M	
*Luzu vaka*	*Ipomoea batatas* (L.) Lamk.	Convolvulaceae	hb/cr	F, M	
*Mahigeli*	*Gulubia hombronii* Becc.	Arecaceae	pl	B	
*Manioko*	*Carica papaya* L.	Caricaceae	tr	F	
*Marihi*	*Dioscorea* spp.	Dioscoreaceae	hb/cl	F	
*Mavuana*	*Flueggea flexuosa* Müll.Arg.	Euphorbiaceae	tr	B, T	BS
*Mokulou*	Unidentified		tr	B	
*Naqarita*	*Cananga odorata* (Lamk.) Hook. f. & Thoms.	Annonaceae	tr-m	M, T	
*Naru*	*Casuarina equisetifolia* J.R. & G. Forst.	Casuarinaceae	tr-m	T	
*Nato* ***n*** *o*	*Rhus taitensis* Guill.	Anacardiaceae	tr	B, T	MS
*Neka*	*Hibiscus manihot* L.	Malvaceae	sh	F	
*Nekete*	*Pipturus argenteus* (Forst. f.) Wedd.	Urticuliaceae	sh/tr-s	M	
*Nobinobi ime*	Unidentified	Fabaceae		T	
***N*** *ohara*	*Cocos nucifera* L.	Arecaceae	pl/tr-m	F, M, B, T	MM
*Nonoqara*	*Nephrolepis hirsutula* (Forst.) Presl	Oleandraceae	fn	T	
*Okete*	*Canarium indicum* L.	Burseraceae	tr-m	F	
*Okokete*	*Garuga floribunda* Dence.*, Canarium vitiense* A. Grat	Burseraceae	tr/tr-m	B	MR, MS
*Onioni*	*Allium cepa* L. var. *aggregatum* G. Don	Amaryllidaceae	hb	F	
*Opiti*	*Spondias dulcis* Sol. ex Parlk., *Averrhoa carambola* L.			F	
*Ore marihi*	*Manihot esculenta* Crantz	Euphorbiaceae	sh	F	
*Paenapo*	*Ananas comosus* (L.) Merr.	Bromeliaceae	hb	F	
*Paloto*	*Palaquium erythrospermum* Lamk.	Sapotaceae	tr	B	MR, ML
*Pamukeni*	*Cucurbita moschata* (Duch. ex Lamk.) Duch. ex Poir	Cucurbitaceae	hb/cl	F	
*Pate*	*Pandanus tectorius* Park.	Pandanaceae	pl/tr	T	
*Pepa*	*Capsicum annum* L. var. *grossum* Sendt.	Solanaceae	hb	F	
*Pepeo*	*Terminalia brassii* Exell	Combretaceae	tr-l	B	
*Petepete*	*Litsea domarensis* Schmidt	Lauraceae	tr	B	MS, ML
*Petu*	*Bruguiera gymnorhiza* (L.) Lam.	Rhizophoraceae	tr	F, B, T	MM
*Petukele*	*Macaranga fimbriata* S. Moore	Euphorbiaceae	tr	B	
*Pidiki*	*Syzygium onesimum* Merr. & Perry	Myrtaceae	tr	B, T	MP, MR, ML, BP
*Pilasi*	Unidentified			T	
*Pinati*	*Arachis hypogaea* L.	Fabaceae	hb	F	
*Pokopoko*	*Campnosperma brevipetiolata* Volkens	Anacardiaceae	tr-l	B, T	MR, ML
*Pomolo*	*Citrus grandis* (L.) Osbeck	Rubiaceae	tr-s	F	
*Qema*	*Pometia pinnata* Forst. f.	Sapindaceae	tr-m	B, T	MR, MS, BP
*Qoliti*	*Gmelina moluccana* Backer ex K.Heyne	Verbenaceae	tr	T	MS
*Rapa*	*Ficus lancibracteata* Corner	Moraceae	tr	B	MR, BS
*Riqi*	*Pterocarpus indicus* Willd.	Fabaceae	tr-m/tr-l	B, T	
*Ruqupole*	*Ocimum basilicum* L.	Lamiaceae	hb/ssh	(F), T	
*Saladia*	*Lactuca sativa* L.	Asteraceae	hb	F	
*Sasopo*	*Annona muricata* L.	Annonaceae	tr-s	F	
*Sili*	*Capsicum frutescens* L.	Solanaceae	hb/ssh	F	
*Sosoruku*	*Sterculia shillinglawii* Muell.	Sterculiaceae	tr	T	
*Suri*	*Diospyros* sp., *Timonius forsteri* DC		sh/tr-s	B, T	MP, MR, ML, BS
*Suti*	*Saccharum officinarium* L.	Poaceae	gr/hb	F	
*Talo*	*Colocasia esculenta* (L.) Schott.	Araceae	hb	F	
*Tamata*	*Lycopersicon lycopersicum* (L.) Karst.	Solanaceae	hb	F	
*Tatalise*	*Terminalia catappa* L.	Combretaceae	tr-m	M	
*Tita*	*Parinari glaberrima* (Hassk.) Hassk.	Chrysobalanaceae	tr	B	MP, MR, ML
*Titimunuhaha*	*Clerodendrum buchananii* (Roxb.) Walp.	Verbenaceae	sh/tr-s	M	
*Tivativa*	*Syzygium* sp.	Myrtaceae	tr	T	MP
*Toqo* ***n*** *eta*	*Timonius timon* (Spreng.) Merr.	Rubiaceae	tr	M, B, T	
*Tototu*	*Sonneratia caseolaris* (L.) Engl.	Sonneratiaceae	tr	B, T	MM
*Totuana*	*Alstonia spectabilis* R. Br.	Apocynaceae	tr	M, B	MS, BP
*Tovinia*	*Canarium salomonense* Burtt	Burseraceae	tr-m	F	
*Tukituki*	*Macaranga* spp.	Euphorbiaceae	tr	B, T	MS, ML, BS
*Turuto* ***n*** *oro*	*Horsfieldia irya* (Gaertn.) Warb.	Myrtaceae	tr	B	MR, ML, BS
*Uotakuresu*	*Nasturtium officinale* R. Br.	Brassicaceae	hb	F	
*Valo*	*Gonystylus macrophyllus* (Miq.) A. Shaw, *G. megacarpus* C.T. White	Thymelaeaceae	tr	B	MP, MR, MS, ML
*Varu*	*Hibiscus tiliaceus* L.	Malvaceae	tr	T	BS
*Vasara*	*Vitex cofassus* Reinw. ex Bl.	Verbenaceae	tr-l	B, T	BP, BS
*Vasavasara*	*Geniostoma rupestris* J.R. & G. Forst.	Loganiaceae	tr	B	MS, BP
*Vogi*	*Crinum asiaticum* L.	Amaryllidaceae	hb	M	
*Voko*	*Ptychosperma salomonense* Burret	Arecaceae	pl	T	
*Vorusu*	*Ceriops tagal* (Pers.) C.B. Rob.	Rhizophoraceae	tr	B	
*Vosevose*	*Neonauclea* spp.	Naucleaceae	tr	B, T	BP, BS,
*Vuagore*	*Dysoxylum excelsum* Bl.	Meliaceae	tr	T	BS
*Zamara*	*Commersonia bartramia* (L.) Merr.	Sterculiaceae	tr-s	B	MS
*Zovi*	*Premna corymbosa* (Burm. f.) R. & W.	Verbenaceae	tr-s	T	
No name (introduced plant)	*Catharanthus roseus* (L.) G. Don	Apocynaceae	hb	M	
Name unidentified 1				B	
Name unidentified 2				B	
Name unidentified 3				B	
Name unidentified 4				B	
Name unidentified 5				B	
Name unidentified 6				B	
Name unidentified 7				B	

^a^ Plant type: cl = climber, cr = creeper, ep = epiphyte, fn = fern, gr = grass, hb = herb, sh = shrub, tr = tree (size unidentified), tr-s = tree-small (<12 m tall), tr-m = tree-medium (12–25 m tall), tr-l = tree-large (>25 m tall) (Henderson and Hancock [4]).

^b^ Use purpose: F = food, M = medicine, B = building, T = tool; use purposes observed during the research periods are shown; those observed outside of the study period were also shown in parenthesis.

^c^ Forest class: MP = main island, primary forest, MR = main island, reserve forest, MS = main island, secondary forest, ML = main island, logged forest, BP = barrier island, primary forest, BS = barrier island, secondary forest, MM = main island, mangrove; only trees observed in this study were shown.

#### *Plants used to treat illnesses*

Herbal medicines were used in 14.5% and 25.4% of treatments for ill person-days, accounting for 159 and 201 cases in the urban and rural areas, respectively. Nineteen species were used on 112 person-days in the villages combined. Coconut oil, used on 30 person-days to treat wounds and skin conditions or for pain relief massage, was the most common herbal treatment. Coconut oil mixed with oil extracted from ylang-ylang (*Cananga odorata* (Lam.) Hook. f. & Thomson) was used on 25 person-days. Rose periwinkle (*Catharanthus roseus* (L.) G. Don)*,* which was recently introduced as a flowering and ornamental plant in urban areas, was used on 16 person-days for diabetes; no Roviana name was recognized for this plant. Heartleaf hempvine (*Mikania cordata* (Burm. f.) B. L. Rob.) was used to treat wounds. Table 3 shows representative tree or palm species used for treatment. In addition to coconut palm and ylang-ylang, three species (tropical almond, betel-nut palm (*Areca catechu* L.), and great morinda (*Morinda citrifolia* L.)) were used for 2.0% or 2.5% of total ill person-days in the two villages, respectively. More medicinal species were used in the rural areas. Almost all herbal plants were planted in the settlements or grown under semidomesticated conditions in secondary growth nearby. Tropical almond and *Cassia alata* L. (not in the table) are representative semidomesticated species.

**Table 2:** Six tree-like or palm food species that contributed the most to villagers’ energy intakes

**Common name**	**Scientific name**	**Roviana name**	**% total energy intake**	
			**Urban**	**Rural**
Coconut	*Cocos nucifera* L.	*Nohara*	3.9	4.3
Canarium nut	*Canarium indicum* L./*C. salomonense* Burtt	*Okete/tovinia*	0.1	3.9
Banana	*Musa* spp.	*Hakua*	2.2	0.4
Papaya	*Carica papaya* L.	*Manioko*	0.6	0.1
Large-leafed mangrove	*Bruguiera gymnorhiza* (L.) Lam.	*Petu*	0	0.04
Gnetum	*Gnetum gnemon* L.	*Leqe*	0.02	0

#### *Plants used to build houses*

Plants used as building materials comprised 71 species, including trees, palms (including rattan), and bamboo. Table 4 shows the 11 species most frequently used by the households in both villages. The two most frequently used species were *Calophyllum* spp. (100% in each village) and *V. cofassus* (100% and 88.2% in the rural and urban villages, respectively). These trees were too large for the rural people to fell by themselves without chainsaws; therefore, the villagers frequently purchased sawn timber from the logging company campsites. Low prices are available for low-grade timber in the local market. Sago palm (*Metroxylon* spp.) leaves were the major materials for walls and roofs in traditional leaf houses; the palm leaves were tightened with thin sticks made from local areca-nut palm trunks (*Areca macrocalyx* Zipp. ex Blume). Rattan palms (*Calamus* spp.) were used as ropes to tighten joints and many other parts of the houses; iron nails were rarely used in building traditional leaf houses. Even many households living in permanent houses had small kitchen huts in which rattan was used (93.3% and 82.4% in the rural and urban villages, respectively). The medium-sized flueggea tree (*F. flexuosa*) was a major source of posts used to build leaf houses; therefore, this tree was used more in the rural village than in the urban village (93.3% and 70.6%, respectively). This tree was used in a round-log form after removal of its bark. Fijian longan (*P. pinnata*) was frequently used in the urban village (94.1%) and rarely used in the rural one (20.0%). Logs from this tree were produced at the logging campsites and traded in this area; this large tree is grown primarily on the barrier islands. Since logging operations had been prohibited on the barrier islands of the Saikile clan until recently, the timbers of this tree were rarely used in the rural village. Large-leafed mangroves (*B. gymnorhiza* (L.) Lam.), which were more abundant in the rural areas, were more frequently used in the rural village (66.7%) than in the urban village (47.1%). Trees that were abundant in secondary growth (brown kurrajong (*C. bartramia*) and *D. salomonensis*) comprised materials for building leaf houses and were used exclusively in the rural village.

**Table 3:** Nine tree or palm species used in two or more person-days to treat illness

**Common name**	**Scientific name**	**Roviana name**	**Frequency of use: no. of person-days (%)**			
			**Urban**		**Rural**	
Coconut	*Cocos nucifera* L.	*Nohara*	25	(15.7)	5	(2.0)
Ylang-ylang	*Cananga odorata* (Lamk.) Hook. f. & Thoms.	*Naqarita*	25	(15.7)	0	(0)
Tropical almond	*Terminalia catappa* L.	*Tatalise*	0	(0)	5	(2.0)
Betel nut	*Areca catechu* L.	*Heta*	0	(0)	4	(2.0)
Great morinda	*Morinda citrifolia* L.	*Gurata*	4	(2.5)	0	(0)
Timonius timon	*Timonius timon* (Spreng.) Merr.	*Toqoneta*	0	(0)	2	(1.0)
Red clerodendrum	*Clerodendrum buchananii* (Roxb.) Walp.	*Titimunuhaha*	0	(0)	2	(1.0)
Native mulberry	*Pipturus argenteus* (Forst. f.) Wedd.	*Nekete*	0	(0)	2	(1.0)
Key lime	*Citrus aurantifolia* (Christm.) Swing.	*Laini*	1	(0.6)	1	(0.5)

#### *Plants used for tools*

Among the various daily commodities, 27 tools were made from 53 species of plants. Table 5 shows the 13 tree or palm species used most frequently for tools. Vitex (*V. cofassus*) was used in all households in both villages to make paddles, wood mortar, agricultural tools, and furniture. Kapok and *B. malabaricum* fiber were used for pillows in almost all households (86.7% and 100% in the rural and urban villages, respectively). Coconut palm leaves were used for hats, bags, fans, and other items, while sago palm leaves were used for brooms. White beech was one of the most important tree species because canoes are made exclusively from this species (80.0% and 70.6% in the rural and urban households, respectively). A significant difference between the two villages (66.7% in the rural village, 0% in the urban one) was found in the use of large-leafed mangroves, because mangrove forests are abundant only in the rural village. The mangroves were used as sticks or knives to open coconut fruit (*viviguana* in the Roviana language). A difference was also observed (93.3% and 23.5% in the rural and urban villages, respectively) in the use of premna (*Premna corymbosa* Rottler & Willd.) for amulets, including amulets used to fish or ward off devil spirits, for example. This customary charm was less frequently found in the urban village.

**Table 4:** Eleven tree or palm species used in the highest frequencies for building houses

**Common name**	**Scientific name**	**Roviana name**	**Frequency of use: % household using**		**Main purposes**
			**Urban**	**Rural**	
Calophyllum	*Calophyllum* spp.	Buni	100	100	Floor, Wall, Post
Vitex	*Vitex cofassus* Reinw. ex Bl.	Vasara	88.2	100	Floor, Wall, Post
Sago palm	*Metroxylon* spp.	Edeve	82.4	93.3	Roof, Wall
Rattan	*Calamus* spp.	Aroso	82.4	93.3	Rope
Betel nut palm (wild)	*Areca macrocalyx* Zipp. Ex Bl.	Heta pinomo	82.4	93.3	Rafter, Wall, Floor
Flueggea	*Flueggea flexuosa* Müll.Arg.	Mavuana	70.6	93.3	Post
Fijian longan, taun	*Pometia pinnata* Forst. f.	Qema	94.1	20.0	Floor, Wall, Post
Large-leafed mangrove	*Bruguiera gymnorhiza* (L.) Lam.	Petu	47.1	66.7	Post, Rafter
Brown Kurrajong	*Commersonia bartramia* (L.) Merr.	Zamara	0	66.7	Rafter, Beam
Timonius timon	*Timonius timon* (Spreng.) Merr.	Toqoneta	41.2	13.3	Beam
Dillenia salomonensis	*Dillenia salomonensis* (C.T. White) Hoogl.	Kapuhu	0	46.7	Floor, Post

### Distribution of useful trees in different forest types and islands

In the vegetation quadrat surveys conducted on the main island, 168 (31 species), 120 (49), 181 (48), and 180 (49) individual trees (> 10 cm DBH) were found in the four quadrats in the primary, reserve, secondary, and logged forests, respectively (Table 6). In addition, 137 trees (10 species) were observed in the mangrove habitat. On the barrier island, 117 (19 species) and 124 (36) trees were found in the primary and secondary forests, respectively.

**Table 5:** Thirteen tree or palm species used in the highest frequencies for tools

**Common name**	**Scientific name**	**Roviana name**	**Frequency of use: % household using**		**Main purpose**
			**Urban**	**Rural**	
Vitex	*Vitex cofassus* Reinw. ex Bl.	Vasara	100	100	Paddle, Furniture, Mortar, Plow
Kapok	*Ceiba pentandra* (L.) Gaertn., *Bombax malabaricum* DC.	Lozi	100	86.7	Pillow
Coconut palm	*Cocos nucifera* L.	Nohara	100	86.7	Basket, Hat
Sago palm	*Metroxylon* spp.	Edeve	82.4	93.3	Broom
Rattan	*Calamus* spp.	Aroso	88.2	86.7	Chair, Tong
Calophyllum	*Calophyllum* spp.	Buni	76.5	93.3	Spear hand, Furniture
Fijian longan, taun	*Pometia pinnata* Forst. f.	Qema	82.4	80.0	Axe hand
Pemphis	*Pemphis acidula* J.R. & G. Forst.	Bobogele	94.1	60.0	Pestle, Coconut opener
White beech	*Gmelina moluccana* Backer ex K.Heyne	Qoliti	70.6	80.0	Canoe
Pandanus	*Pandanus* sp.	Dalou	41.2	80.0	Mat
Premna	*Premna corymbosa* Rottler & Willd.	Zovi	23.5	93.3	Amulet
Pandanus tectorius	*Pandanus tectorius* Park.	Pate	29.4	40.0	Mate
Large-leafed mangrove	*Bruguiera gymnorhiza* (L.) Lam.	Petu	0	66.7	Pestle, Coconut opener

**Figure 3:**
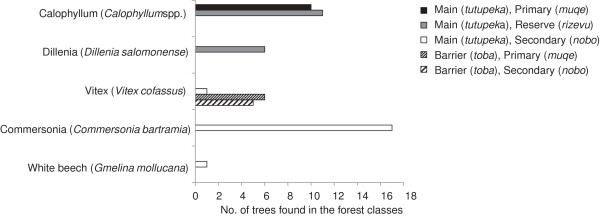
Number of selected useful trees in each forest class.

The number of trees observed in the plant use survey quadrats was 87 (15 species), 82 (26), 53 (18), 80 (12), and 77 (16) in the main island’s primary, reserve, and secondary forests and the barrier island’s primary and secondary forests, respectively. A total of 87 trees (6 species) were found in the mangrove quadrats (Table 6). The proportion of useful trees was high in the barrier island’s primary forest (68.4%) and the main island’s reserve (68.3%) and low in the main island’s secondary forest (29.3%). Figure 3 shows the numbers of trees of representative useful species found in the respective forest use classes. Calophyllum (*Calophyllum* spp.), a primary building material, was predominantly found on the main island and in primary and reserve forests. Dillenia (*D. salomonensis*) was found only in the reserve forests. Commersonia (*C. bartramia*), an important building material in the rural village, and white beech, a rare species, were found only in the secondary forests. Vitex (*V. cofassus*), an important building and tool species, was found on both islands, although it was more frequently observed on the barrier islands. These results suggest that each forest class has a different level of importance to the peoples’ subsistence lifestyle.

Table 7 shows the similarities between the different types of vegetation. The primary forests on the main and barrier islands were only half as similar (16.0%) as the respective secondary forests (33.3%). Vegetation in the reserve forests was 45.9–52.6% similar to that found in the primary, secondary, and logged forests on the main island but less similar to the primary and secondary forests on the barrier island (17.4–33.8%). Species found in the mangrove forest were not observed in other forest classes. These results suggest that each forest class represented a different vegetative community, with relatively low rates of similarity between communities. Assuming that primary forest represented areas with little human impacts, several unique species were found in human-modified forests (i.e., reserve forest and secondary forests). The vegetation on the main and barrier islands was very different.

**Table 6:** The numbers of trees, tree species, and proportions of useful trees found in each forest class

	**No. of trees (per 1/4 ha)**	**No. of species (per 1/4 ha)**	**No. of useful trees (per 1/4 ha)**	**No. of useful species (per 1/4 ha)**	**% of useful trees**	**% of useful species**
Main island (Tutupeka)						
Primary	168	31	87	15	51.8	48.4
Reserve^a^	120 (122, 117)	49 (27, 41)	82	26	68.3	53.1
Secondary	181	48	53	18	29.3	37.5
Mangrove	137	10	87	6	63.5	60.0
Logged (selectively)	180	49	92	20	51.1	40.8
Barrier island (Toba)						
Primary	117	19	80	12	68.4	63.2
Secondary	124	36	77	16	62.1	44.4

^a^Two quadrats (1/4 ha each) were made and the number of trees were averaged for the reserve (the numbers of trees and species for respective quadrats were shown in the parentheses).

**Figure 4:**
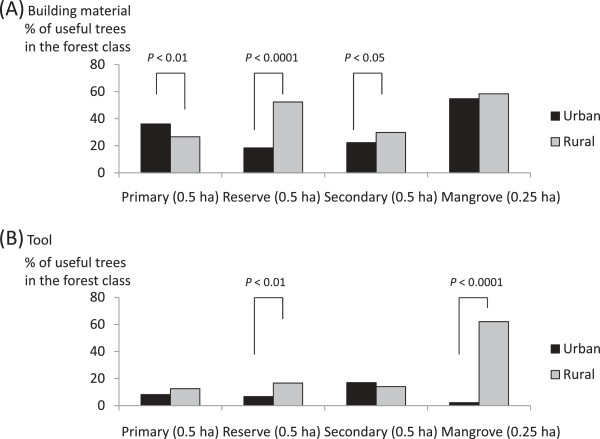
Comparison of proportions of useful trees (used as building materials (A) and tools (B)) in each forest class between urban and rural.

Figure 4 shows the proportions of plants used for building materials and tools in the different forest classes by the urban and rural people. Primary forest trees used as building materials accounted for 36.0% of the urban village tree species and only 26.6% of the rural village tree species (*χ*^2^ = 7.7, *P* = 0.004); however, most of the building materials in the former group were timbers purchased from logging companies operating in neighboring areas. In the reserve forests, 52.3% of the trees were species used by the rural people as building materials; this proportion was only 18.4% in the urban village (*χ*^2^ = 58.6, *P* <0.001). Trees found in the mangrove forests were seldom used to make tools in the urban village, where few mangroves remained, while 62.3% of the tree species in the mangroves were used in the rural village. The reserve forests had more useful tree species in the rural (16.7%) village than in the urban village (6.7%; *χ*^2^ = 10.7, *P* = 0.001). These results suggest that the reserve and mangrove forests, which existed exclusively in the rural village, provided the rural people with a number of useful resources that the urban people did not have access to.

In the urban village, materials that were not available from forests were purchased. For instance, large-leafed mangrove sticks were used to pry open coconuts (*viguvigua* in Roviana) in nine of 15 households in the rural village, while imported iron was used for this purpose in the urban village.

## Discussion and conclusions

### Subsistence use depends on the botanical diversity in human-modified forests

This study documented how the Roviana people traditionally use each of several forest types and how each forest type supports different plant species that are used for various purposes. We examined four use categories of plants: food, medicine, building materials, and tools (Tables 2, 3, 4 and 5, respectively). Coconut was represented in all four use categories, suggesting that this plant is the most important species for the Roviana people. Coconut is important in Pacific Island societies because it provides a high-calorie source of food, building materials, herbal medicines, and fuel [4,5,43]. A mangrove species (*B. gymnorrhiza*) was used in three (foods, building, and tools) of the four use categories. According to a previous study, mangroves also have various purposes (i.e., food, medicine, building materials, tools, firewood) in Marovo [5], and mangroves are believed to have traditionally played important roles in the subsistence of the Western Solomon Islanders. *Timonius timon* (Spreng.) Merr., which is not commonly used in other societies, was also used in three categories, i.e., medicine, building materials, and tools. This tree grows wild on cleared land and roadsides. The people weed its seedlings in their gardens or plantation areas but leave it untouched elsewhere; in addition, they sometimes cut/weed trees that might harm nearby timonius trees to help timonius tree growth. Such behaviors, called “semidomestication” [47,48], were observed with other plants, such as gnetum and premna. The villagers thus enjoyed a range of ecological services provided by the biodiversity that was preserved in their forests.

### Traditional ways of subsistence may contribute to forest biodiversity

The biodiversity of the landscape was related to the people’s use and management of forest resources. We found that the human-modified reserve and secondary forests represented distinct vegetative communities that are very different from both the primary forest, which has experienced few human impacts, and from one another (Tables 6 and 7, Figure 3). For example, some pioneer species (e.g., *D. salomonense*) grew only in forest gaps in the reserve forest and not in mature forest; these gaps were created by the regular cutting of useful trees by the villagers (which was permitted, although commercial use or clearance for gardens was not) for their subsistence. Others (e.g., *C. bartramia* and *G. mollucana*) occurred only in secondary forest (Figure 3, Table 7), which regrew after shifting cultivation crop fields were abandoned when productivity decreased. In addition, semidomestication is also thought to have promoted the growth of specific species, such as timonius, which are uncommon in the wild but sometimes encouraged by the villagers when they do occur [47,48]. Thus, regular subsistence use of the forests by humans may have resulted in multiple unique vegetative communities, and higher overall biodiversity, than would otherwise exist. Alternatively, the diverse vegetative communities may have attracted the people, but this hypothesis is contradicted by the small number of forest types near the more populous urban village. The logical conclusion is that the existence of a variety of forest classes, i.e., human-modified forests, both increases diversity and provides an essential base for the rural people’s subsistence.

### Socioeconomic changes can affect diversity, even in ecologically-autonomous communities

In Dunde village, biodiversity has already been lost. The reserve and mangrove forests no longer exist or are severely diminished. Consequently, the urban people did not enjoy the ecological services of useful plants that would be expected in those habitats. For example, the mangrove has been an important useful plant in this area (see above), but it was rare in the urban areas, and its rate of use was low. The Dunde villagers were required to purchase other tree resources using cash or use modern materials to compensate for this lost biodiversity.

**Table 7:** Sørensen similarity index (%) between different forest classes

	**Main island**				**Barrier island**	
	**Reserve**	**Secondary**	**Mangrove**	**Logged**	**Primary**	**Secondary**
Main						
Primary	49.5	27.8	0	40.0	16.0	14.9
Reserve		45.9	0	52.6	17.4	33.8
Secondary			0	49.0	20.9	33.3
Mangrove				0	0	0
Logged (selectively)					21.9	31.1
Barrier						
Primary						40.0

In recent years, under an expanded market economy, widespread commercial logging has been carried out, and industrial reforestation is now being implemented in logged areas. This trend, if it continues, could destroy the residents’ source of livelihood, triggering a decrease in the number and variety of forest species. Although industrial timber plantations will contribute to increased biomass, they may interrupt the original cycle of biodiversity and disrupt the relationships between humans and forests. People will lose access to vital ecosystem services from their range of forest classes if logging and cash crop planting continues unabated [23,49]. Building timber plantations will not replace the current ecosystem benefits. Previous studies have suggested that shifts from subsistence agriculture to cash crops have adversely affected labor, the economic status, and natural productivity in other parts of Solomon Islands [5,19,40,49,50]. In contrast, a recent monetary study reported that villagers were willing to pay a high proportion of their incomes to conserve ecosystem services [51]. Thus, efforts to conserve forest biodiversity may also economically benefit the Roviana people.

### Biodiversity conservation programs can integrate local peoples’ modifications of forests

#### Local people possess vital conservation knowledge

**Figure 5:**
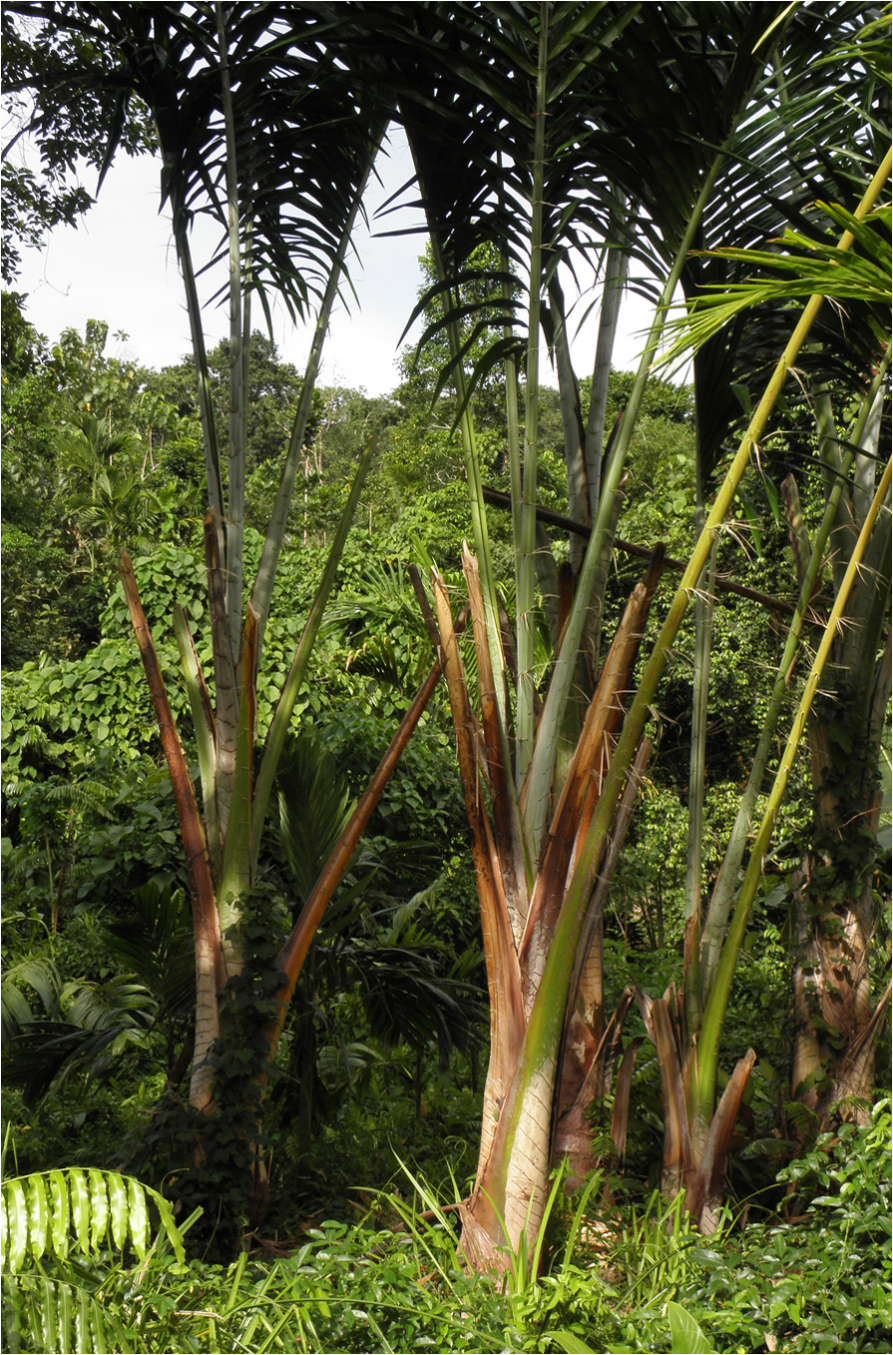
Sago palm with four leaves remaining after the villagers collected the other leaves.

Throughout this study, the rural people conserved various forest types and thus preserved the diversity of tree species while enjoying the benefits of the forests in their territories. There are customary rules about forest conservation in Olive, such as for the use of a canoe-making tree, the white beech. When a person needs a new canoe, he/she must find a young white beech tree and mark it to inform the other residents of its intended use. Then, when the time comes to make the canoe, he/she must ask for the customary chief’s permission. This tree is also grown on cleared land [3,39]; therefore, human modification contributes to the sustainability of this resource. There is also a rule for gathering sago palm (*Metroxylon* spp.) leaves, an essential roof and wall material in traditional houses: because a sago palm takes a very long time to regrow once the trunk is cut, the trunk must be left untouched and only the leaves can be removed. Four leaves should also remain on the tree, because it will die if all the leaves are removed (Figure 5). If a villager violates this rule, he/she will be penalized by the chief (e.g., compensation payment). According to the interviewees, the rural people established the reserve forest in the 1970–80s based on the recommendation of the leader of the CFC and the chief; this was because they intended to expand a coconut plantation for cash income and the logging operation had been coming near their territory. These cases suggest that local leadership, especially from the chief and church leaders, and traditional ecological knowledge are important for conservation.

### Traditional forest uses can enhance biodiversity conservation and quality of life

Local leaders play important roles in the conservation of biodiversity. However, a dilemma now exists because these leaders are also leading rural development initiatives aimed at improving the quality of people’s lives [46]. The same situation exists at the national level. Generally, the governments of the Pacific Island countries depend on the production of natural resources for revenue. In addition, even if a government tries to take action related to natural resource conservation, successful outcomes are difficult to achieve because most territories consist of lands with customary uses designated by the local people; a group of land owners may not agree on conservation but might accept development projects [6]. From the viewpoint of biodiversity conservation, the rural residents of Solomon Islands, whose livelihoods depend heavily on farming and fishing, have no choice but to continue making a living using natural resources. Even for the sake of conserving natural resources, it is virtually impossible to persuade people to leave unmodified forests untouched. Even if such ideas were accepted, the agreements are unlikely to become permanent.

Recent studies have suggested that, worldwide, there exist few pristine natural areas that are free from any human impacts [52]. Top-down efforts to conserve pristine environments, with a few exceptions, have failed, so that recent conservation focus has shifted to new paradigm of incorporating a productive landscape, social institutions, and human-modified forests [12,13,53]. Conservation activities are also more efficient if conservation forests and production lands are separated, but tropical societies [54], as in this study, depend strongly on their forests for production. Conservation efforts, either community-based or by outsider’s initiatives, have to allow for human uses of forests rather than trying to protect pristine environments, such as in the zone-based conservation model [55]. The findings of this study suggest that conservation of virgin forest is not acceptable to the Roviana people, who live in human-modified forests, and may diminish biodiversity. Therefore, the focus of conservation must shift toward human-modified forests where the people use the natural resources in a sustainable way, as in the Satoyama Initiative in the CBD [16-18]. However, this human–environment relationship is easily transformed during socioeconomic changes.

## Conclusions

We identified four major limitations of our study. First, we only interviewed four landscape experts (although this number compared favorably with previous studies [10]). Knowledge regarding the forest varied within the community; e.g., the younger generations were less informed. Therefore, we used only forest experts that were recommended by village leaders and who were respected in the villages for their traditional ecological knowledge. These experts rarely disagreed with one another on forest knowledge. Indigenous people of Solomon Islands and other Melanesian societies know their vegetative communities [5,11]. We thus believe that our informants were representative of the villagers’ knowledge of landscape classification.

A second limitation was logistic; we could not feasibly survey all recognized vegetation classifications. We choose 15-year-old fallow areas to represent secondary forests because forests of this age existed on both the main and barrier islands, and the villagers could accurately identify the age by referring to important events. Other forest types, such as *hope* and *emata,* were small or difficult to distinguish from other forest classes (i.e., ‘primary’ or ‘secondary’ forests). While this study may not have revealed all of the vegetative differences at the study sites, our data well represented the diversity of forest uses and revealed clear differences between the urban and rural villages.

Third, to protect the villagers’ intellectual property and privacy, we did not record some kinds of plant uses, such as magical purposes and medicines. Fourth, our observations were restricted to a season and may not represent all forest uses throughout the year. However, compared with our previous work in these villages and with others studies of neighboring areas [4,5], our results included most human physical effects on forest trees.

The findings of this study from the urban village suggested that the Roviana people can also maintain their lifestyle without the ecological services provided by biodiversity, although much effort is required to earn cash as alternatives to reserves and mangroves. However, this model is not sustainable, because, as observed in our previous study, approximately one-third of the urban villagers earn the same amount of cash as the rural villagers [33]. In addition, the purchased materials are produced from logging operations in rural forests [15,18]. Therefore, it may be necessary to provide economic incentives to conserve human-modified forests, i.e., conserving biodiversity while using ecological services. In addition, a loss of economic use, e.g., the fact that mangroves are now a low-priority resource and at risk of disappearing in urban areas, may lead to loss of traditional knowledge and cultural diversity. Our results highlight the importance of human-modified forests in forest conservation initiatives, such as REDD + (reducing emissions from deforestation and forest degradation in developing countries and the role of conservation, sustainable management of forests and the enhancement of forest carbon stocks in developing countries) or PES (payment for ecosystem services).

## Competing interests

The authors declare that they have no competing interests.

## Authors’ contributions

TF designed the study. TF, MS, and RO carried out the field research. MQS identified the scientific names of the plant specimens. RO supervised the work. TF performed the statistical analyses. TF, MQS, and MS analyzed the data and wrote the manuscript. All authors read and approved the final version of the manuscript.
